# An Innovative Procedure for Calibration of Strapdown Electro-Optical Sensors Onboard Unmanned Air Vehicles

**DOI:** 10.3390/s100100639

**Published:** 2010-01-18

**Authors:** Giancarmine Fasano, Domenico Accardo, Antonio Moccia, Attilio Rispoli

**Affiliations:** 1 Department of Aerospace Engineering, University of Naples “Federico II”, P.le Tecchio 80, 80125 Naples, Italy; E-Mails: domenico.accardo@unina.it (D.A.); antonio.moccia@unina.it (A.M.); 2 Aeronautical Systems Division, Italian Aerospace Research Center, Via Maiorise, 81043 Capua (CE), Italy; E-Mail: a.rispoli@cira.it

**Keywords:** electro-optical sensors, alignment, boresighting, carrier-phase differential GPS, attitude and heading reference system, target pixel extraction, Q-method

## Abstract

This paper presents an innovative method for estimating the attitude of airborne electro-optical cameras with respect to the onboard autonomous navigation unit. The procedure is based on the use of attitude measurements under static conditions taken by an inertial unit and carrier-phase differential Global Positioning System to obtain accurate camera position estimates in the aircraft body reference frame, while image analysis allows line-of-sight unit vectors in the camera based reference frame to be computed. The method has been applied to the alignment of the visible and infrared cameras installed onboard the experimental aircraft of the Italian Aerospace Research Center and adopted for in-flight obstacle detection and collision avoidance. Results show an angular uncertainty on the order of 0.1° (rms).

## 1. Introduction

The growing use of Unmanned Air Vehicles (UAVs) in both military and civil scenarios [1,2] requires imaging systems providing adequate performance in terms of Field Of View (FOV), field of regard and geometrical resolution, which affect achievable mission performance. High resolution Electro-Optical (EO) cameras are of special interest because of the very accurate line-of-sight orientation estimation in the sensor FOV that they can provide. This information is very important when a high level of situational awareness is required as in the case of UAV flights in the civil airspace. Worldwide research is on-going concerning UAV “Detect, Sense, and Avoid” (DSA), that is, the capability of unmanned aircraft to detect non cooperating air traffic, to estimate the collision potential and, in case of necessity, to perform safe collision avoidance as in manned flight [3–6]. The installation of a sensor system for autonomous obstacle detection and tracking has been highlighted as mandatory to attain levels of safety equivalent to the ones of manned aircraft during visual flight phases. Indeed, the most appropriate configuration of EO sensor in order to attain this function is a strapdown and forward looking installation.

Besides angular resolution, EO sensors have some interesting peculiarities for DSA such as fast scan rates (on the order of 10 Hz or more), low cost, small size and weight, and since they are passive sensors, low electric power consumption. In addition, the adoption of InfraRed (IR) EO sensors also permits night operation. Thus, there is a flurry of research about the use of EO sensors as obstacle detection sensors which basically follows two lines of reasoning. The first is to use cameras alone with a particular emphasis on image processing algorithms such as optical flow [7]. The second approach is to integrate them with microwave sensors in order to compensate for single sensor shortcomings [8]. In the latter case, the accurate angular information can be used to improve radar-based tracking performance and thus the reliability of the entire sensor system at low distances from the intruders. Moreover, system performance benefits of the EO system data rate that is higher than in the microwave one.

Increased situational awareness is also very important in manned flight, e.g. in the approach and landing phases under bad visibility conditions. In these cases Enhanced Vision Systems (EVS) relying on weather-penetrating forward-looking sensors can augment the naturally existing visual cues in the environment and provide a real-time image of prominent topographical objects that may be identified by the pilot. As in the see and avoid case, these systems are at prototype level and typically integrate infrared (IR) cameras as auxiliary sensors [9].

In all the above considered cases, the accuracy of EO information is of great importance. Besides being dependant on the sensor resolution, overall angular error is also due to mounting errors which introduce angular biases. In these applications, it is worth noting that alignment error refers to the error computed with respect to the body reference frame as individuated by the attitude heading reference system (AHRS). In fact, in the UAV case, navigation data as provided by the AHRS are used for autonomous flight by the flight control computer. Also in the case of manned flight, there exist regulations that prescribe high accuracy alignment of the inertial unit with respect to the aircraft body axes [10].

Indeed, in general the alignment process of strapdown units is addressed with the term “boresighting” [11]. It applies to a wide category of hardware such as Inertial Navigation Systems, weapons, guns, Forward Looking Infra-Red cameras, Head Up Displays, and Air Data Sensors.

Several methods can be used for EO sensors boresighting, based on interferometric, mechanical, or image processing techniques [12–14]. For example, a stereoscopic couple can be calibrated on the basis of the analysis of a sample pattern which allows evaluation of relative translation and rotation, which are the extrinsic parameters that are calculated [15]. However, traditional methods or algorithms are difficult to use to provide calibration of airborne EO sensors directly with respect to the navigation unit.

This paper illustrates a fast and accurate procedure to provide boresighting of EO sensors for UAVs, taking advantage of on-board AHRS attitude measurements, that are very accurate in static mode, and GPS position measurements in carrier-phase differential Real Time Kinematic (RTK) mode. Indeed, carrier-phase receivers are not actually used for UAV navigation, but they are often available during system testing phases, hence they are used as reference for estimating standard navigation measurement accuracy. The proposed technique allows an entire set of EO sensors to be aligned together and can be also used to put in evidence possible effects of flight dynamics on camera mounting. Internal calibration of each camera has to be performed before application of the procedure, in order to evaluate the parameters to be used in the pinhole camera models adopted to describe cameras’ geometry. Main advantages over alternative techniques, such as interferometric ones, can be summarized in the following bullets:

- The proposed method works in end-to-end configuration by exploiting images acquired using EO sensors and AHRS attitude measurements. Indeed, interferometric techniques perform alignment between reference surfaces installed on sensor chassis;
- Alignment setup configuration does not require complicated facilities to be build up such as the optical paths that are needed to carry out interferometric measurements.

The developed method has been applied to align the EO sensors for object detection and collision avoidance on board an optionally piloted aircraft of the Italian Aerospace Research Center (CIRA) in the framework of the TECVOL (Technologies for Autonomous Flight) project [16].

First of all, the alignment procedure is illustrated in detail pointing out both theoretical aspects and practical issues. Subsequently, the paper describes the hardware setup (navigation and EO sensors) which was used to test the alignment technique. Finally, results achieved during a calibration session are illustrated. Capabilities and limitations of the designed procedure, as well as the lessons learned from experiments, are analyzed in the conclusions.

## 2. Procedure Description

The procedure described in this section allows for the simultaneous alignment of all EO sensors installed aboard an aircraft, regardless of the baseline among them and of their operating wavelengths. It requires that at least two images of a target be acquired by all the cameras, while at the same time aircraft attitude is measured by the AHRS and target position is measured by carrier phase differential GPS (CDGPS) in RTK mode. Cameras’ positions must be measured by CDGPS with the same level of accuracy.

Carrier phase position measurements are the most accurate ones that can be performed using the GPS signal [17,18]. In particular, root mean square (rms) accuracy can be on the order of 2–4 mm. As in all differential GPS modes, in the RTK technique GPS signal corrections are transmitted in real time from a reference receiver at a known location to another receiver. This allows one to compensate for atmospheric delay, orbital errors and other variables in GPS geometry, thus increasing positioning accuracy. RTK produces the most precise GPS positioning since it uses the code phase of GPS signals as well as the carrier phase, which delivers the most accurate GPS information, to provide differential corrections.

A base GPS antenna is needed as a reference for calculating in real time corrections for the target receiver. This antenna can be the GPS antenna located on the aircraft, if an onboard receiver with carrier phase mode enabled is available.

In this application, the base antenna position in the Earth-Centered Earth-Fixed (ECEF) reference frame adopted by GPS is not known with high accuracy, but this has no impact on the procedure. In fact, in any case RTK technique allows computation of target position with respect to the reference base antenna at millimeter-level accuracy, and absolute errors in computing reference (and target) position have no effects since the input for the alignment calculation is the relative position of the target with respect to the cameras. The simplest way to accurately measure target position is to use another GPS antenna as the target to be viewed. Of course, it has to be detectable in all the considered images.

In a practical case, it is important to establish how many target positions are to be measured and at what distance the targets must be placed. From a statistical point of view, assuming an uncorrelated random error in computing target line of sight in each observation, a large number N of target positions allows pointing estimation accuracy to be improved on the basis of a N^−0.5^ factor. However, in a practical case stability of GPS estimates can vary from one measurement acquisition to another, so that fewer measurements all taken with the highest accuracy produce a better pointing accuracy.

To evaluate target distance both CDGPS accuracy and sensors’ Instantaneous Field of Views (IFOVs) must be taken into account. Theoretically the best solution would be to place the target as far as possible from the sensor so that GPS error falls well below single pixel linear dimension. However, this may make target identification and positioning within the image harder to carry out. Furthermore, in order to have a globally accurate alignment, the test points should be selected uniformly spaced in the cameras FOVs. In the procedure the target distance has been set at the point where GPS rms accuracy equals the linear dimension corresponding to the cameras IFOV.

In order to determine the rotation matrices between sensors’ reference frames and aircraft Body Reference Frame (BRF) (X-nose, Y-right wing, Z-down) a least squares technique has been adopted (the q-method) which estimates for each camera the transformation matrix on the basis of a series of vector observations of the same points in the two reference frames [19]. In what follows, for the sake of simplicity, body reference frame will be considered coincident to the AHRS-defined reference frame. This frame is also considered as the reference for the Flight Control System installed onboard the CIRA manned laboratory aircraft equipped for automatic control that is named Flying Laboratory for Aeronautical Research (FLARE).

The basic assumption of the q-method is that the main component of the error for a single observation is random. To this aim, intrinsic calibration must be performed for each EO sensor by imaging a sample planar pattern from different points of view [15] which allows estimation of the intrinsic parameters of the classic pinhole camera model [20]. For example, in the considered case a 4th order “plumb bob” model was assumed [15] which suffices for alignment requests also because of the narrow field of view of the cameras. On the other hand, the validity of this assumption can be verified by analyzing the residual errors after camera alignment, which is reported in the following.

**Figure 1.**
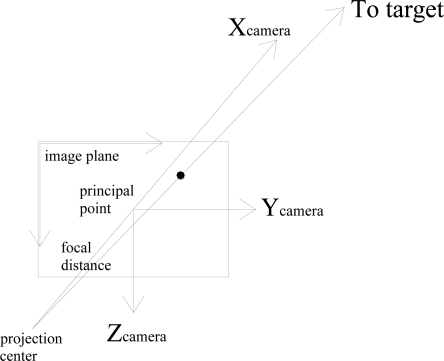
Frontal pinhole camera model (neglecting optical distortions).

Image analysis allows target centre pixel to be identified in each image, then its coordinates can be translated into angular information by exploiting the camera intrinsic parameters. Equivalently, this means that antenna unit vector components in the camera reference frame are estimated in each image. Considered geometry is depicted in Figure 1, where the image plane is represented in front of the projection center, in order to avoid sign inversion (frontal pinhole camera model [20]), and, for the sake of simplicity, optical distortions are neglected.

On the other hand, given the target and the camera position in the ECEF reference frame, it is possible to evaluate target position with respect to the North East Down (NED) reference frame with origin in the camera through an exact transformation if the Earth model is known [21]. In the considered application the WGS-84 ellipsoidal model can be used [22] and deflection of vertical (on the order of 0.03°) can be neglected, since the consequent target position estimation error falls well below GPS accuracy.

Then, the target position *r_iNED_* can be transformed from the camera-based NED to the BRF (again, with origin in the considered camera), on the basis of the attitude angles measured by the AHRS, by Equation (1):

(1) $${\underline{r}}_{\mathrm{iBRF}}=M_{321}\left(\gamma ,\;\beta ,\;\alpha \right){\underline{r}}_{\mathrm{iNED}}$$

where γ, β and α are, respectively, the heading, pitch and roll angle, and the matrix M_321_ is obtained as follows:

(2) $$M_{321}=\;\left[\begin{array}{ccc} \text{cos }\beta \;\text{cos}\;\gamma & \text{cos}\;\beta \;\text{sin}\;\gamma & -\text{ sin}\;\beta \\ -\text{cos}\;\alpha \;\text{sin}\;\gamma +\text{sin}\;\alpha \;\text{sin}\;\beta \;\text{cos}\;\gamma & \text{cos}\;\alpha \;\text{cos}\;\gamma +\text{sin}\;\alpha \;\text{sin}\;\beta \;\text{sin}\;\gamma & \text{sin}\;\alpha \;\text{cos}\;\beta \\ \text{sin}\;\alpha \;\text{sin}\;\gamma +\text{cos}\;\alpha \;\text{sin}\;\beta \;\text{cos}\;\gamma & -\text{sin}\;\alpha \;\text{cos}\;\gamma +\text{cos}\;\alpha \;\text{sin}\;\beta \;\text{sin}\gamma & \text{cos}\;\alpha \;\text{cos}\;\beta \end{array}\right]$$

By dividing *r_iBRF_* by its modulus, it is possible to evaluate the cosine directors of the line-of-sight to the target relevant to the considered camera and the i-th image. Let us call *r̂_iBRF_* the computed unit vector, whereas *r̂_iSENS_* is the unit vector representing the direction to the target in the camera based reference frame as extracted from the i-th image. It is worth noting that camera reference axes have been chosen here with the same convention of the aircraft BRF (X nose-Y right-Z down).

It is now possible to define a loss function:

(3) $$J(M_{\mathrm{CAM}})=\sum_{i=1}^{n}\;w_{i}\;{\left|{\underline{\hat{r}}}_{\mathrm{iSENS}}-M_{\mathrm{CAM}}\;{\underline{\hat{r}}}_{\mathrm{iBRF}}\right|}^{2}$$

where n is the number of collected images/positions, w_i_ is the weight of the i-th measurement and M_CAM_ is the attitude matrix of the considered camera with respect to the aircraft. The loss function is thus the weighted sum squared of the difference between the measured and transformed vectors. An optimal choice of M_CAM_ is that which minimizes J. It can be computed by means of the algorithm named q-method which calculates attitude in terms of the corresponding optimal least-square quaternion. The q-method is a standard technique for estimation of satellites’ attitude based on star sensor measurements. A detailed demonstration of the least-square method and a description of the algorithm can be found in [19]. In the considered case, all the measurements have the same weight.

Alignment accuracy is strongly dependant on the stability of AHRS and GPS estimates. Thus, a critical point is the control of attitude angles drift to ensure that no anomalous oscillation is observed, and the control of GPS estimated accuracy while acquiring targets and cameras positions. Assuming an internal calibration with sub-pixel level accuracy and an accurate extraction of target pixels, these two factors are the most important in determining resulting accuracy.

An important aspect to be taken into account is that AHRS systems measure heading angle with respect to the magnetic North, while the transformation in equation from ECEF to NED refers to the geographic North. Thus, AHRS heading must be referred to geographic North by summing magnetic declination [23]. In case of ignored or non correct estimation of magnetic declination this would introduce a systematic error in alignment.

The illustrated procedure extracts the rotation matrices between the cameras and the AHRS reference frame. It must be considered that the parallax error due to the distance among EO sensors and AHRS must be taken into account in order to convert, in flight, EO estimates to the BRF with origin in the inertial unit. Indeed, for a given camera, the parallax effect can be neglected or not depending on the distance of a target: when using EO data for real time tracking, this error can be corrected on the basis of the estimated range.

**Figure 2.**
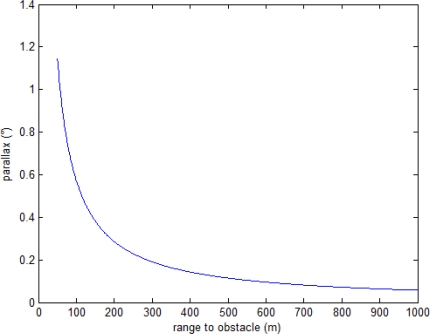
Parallax effect as a function of range to obstacle for a 1 m separation between camera and AHRS.

For example, if the vertical separation between cameras and AHRS is about 1 m parallax contribution is remarkable only at very small ranges to obstacle (Figure 2), on the order of the bubble distance that is the minimum separation between aircraft that must be guaranteed (about 160 m).

## 3. Hardware Setup

The sensor system prototype for DSA has been initially installed on-board the FLARE aircraft. The project aims at verifying by flight tests the adequacy of attained performance for supporting fully autonomous flight. The anti-collision sensor system is based on real time fusion of radar and EO data and is illustrated in detail in Reference [8]. The EO system is comprised of two visible high resolution cameras (panchromatic and color) and two thermal IR cameras. The two visible cameras have the same optics and are installed parallel to the aircraft longitudinal axis to get simultaneously a high resolution panchromatic image and a color one of the same region. Due to their limited angular aperture, the two IR cameras during collision avoidance tests are pointed slightly eccentric to get a field of view comparable to visible cameras. In particular, the visible cameras are the Marlin™ F145B2™ and F145C2™, produced by Allied Vision Technologies GMBH™ (AVT). Both communicate via an IEEE1394 IIDC interface and are capable of producing color/panchromatic images up to 1,392 × 1,040 pixels. They were equipped with MV618T™ optics realized by AVT with focal length of 6.5 mm and thus a field of view (FOV) of 52.9° × 40.8°. The IR cameras are the A40V ™ produced by FLIR Systems™, with a resolution of 320 × 240 pixels and a field of view of 24° × 18°.

As for navigation sensors, the central unit is the AHRS400CC™ manufactured by Crossbow™ which is a solid-state attitude and heading reference system. In static mode it is possible to eliminate uncorrelated noise by averaging sensors output for some seconds. Moreover, if attitude error biases are properly estimated in the AHRS calibration process [24], they can be almost completely removed and resulting attitude measurement accuracy in static conditions is on the order of 0.1°.

The ground GPS antenna used to test the alignment technique is the LegAnt™ manufactured by Topcon™, whereas other two GPS antennas are located on the aircraft wings. As already stated one of these antennas is used as reference for the RTK differential GPS mode. The relevant accuracies for procedure implementation are shown in Table 1.

The distance from the target can be individuated as the one where GPS accuracy equals the linear dimension which corresponds to the cameras IFOV. Some numerical data for the considered case are shown in Table 2.

**Figure 3.**
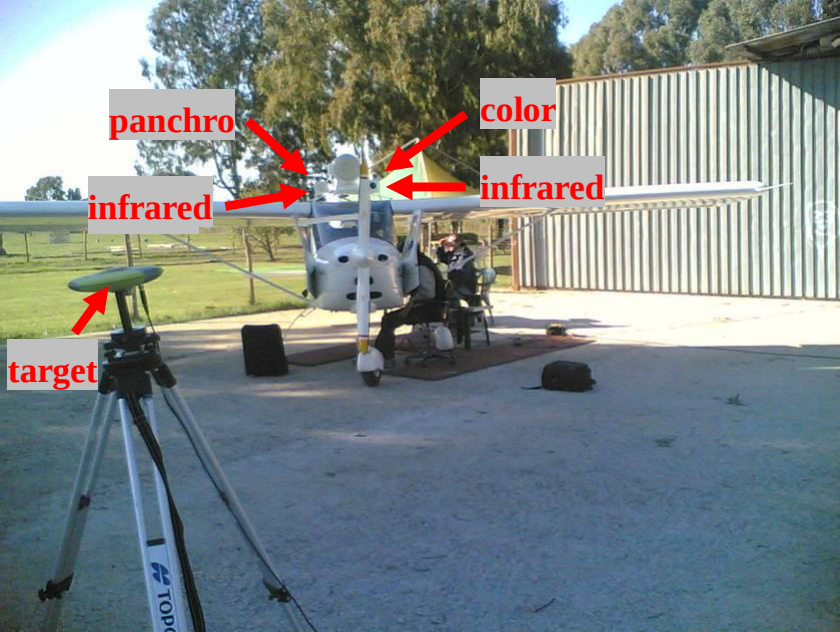
Calibration procedure: acquisition of target images and position.

Table 2 shows that in the considered case the procedure can be implemented by locating the target at a distance of about 4 meters from the focal plane of the sensors and moving it in a rectangle of about 4 m by 3 m. Thus, this distance was selected in the performed tests. Figure 3 clarifies sensors’ installation onboard the aircraft and shows part of the hardware set-up used during a calibration session.

## 4. Experimental Results

Alignment sessions were performed during experimental activities connected with sense and avoid flight tests. Some results relevant to the same alignment session are summarized in the following. For the sake of brevity, only one of the infrared cameras is considered.

**Figure 4.**
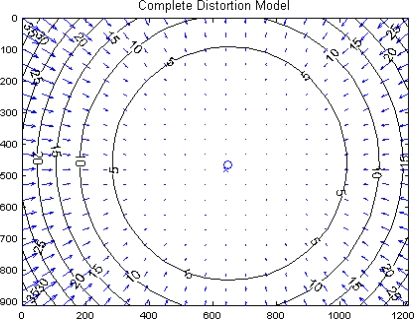
Estimated distortion model for the panchromatic camera (pixel units along the axes).

As already stated, before installation onboard the aircraft, sensor internal calibration has been performed. As an example, the panchromatic camera optical distortion was estimated to be mostly radial with an effect on the order of 25–30 pixels at the limits of the FOV, as shown in Figure 4.

**Figure 5.**
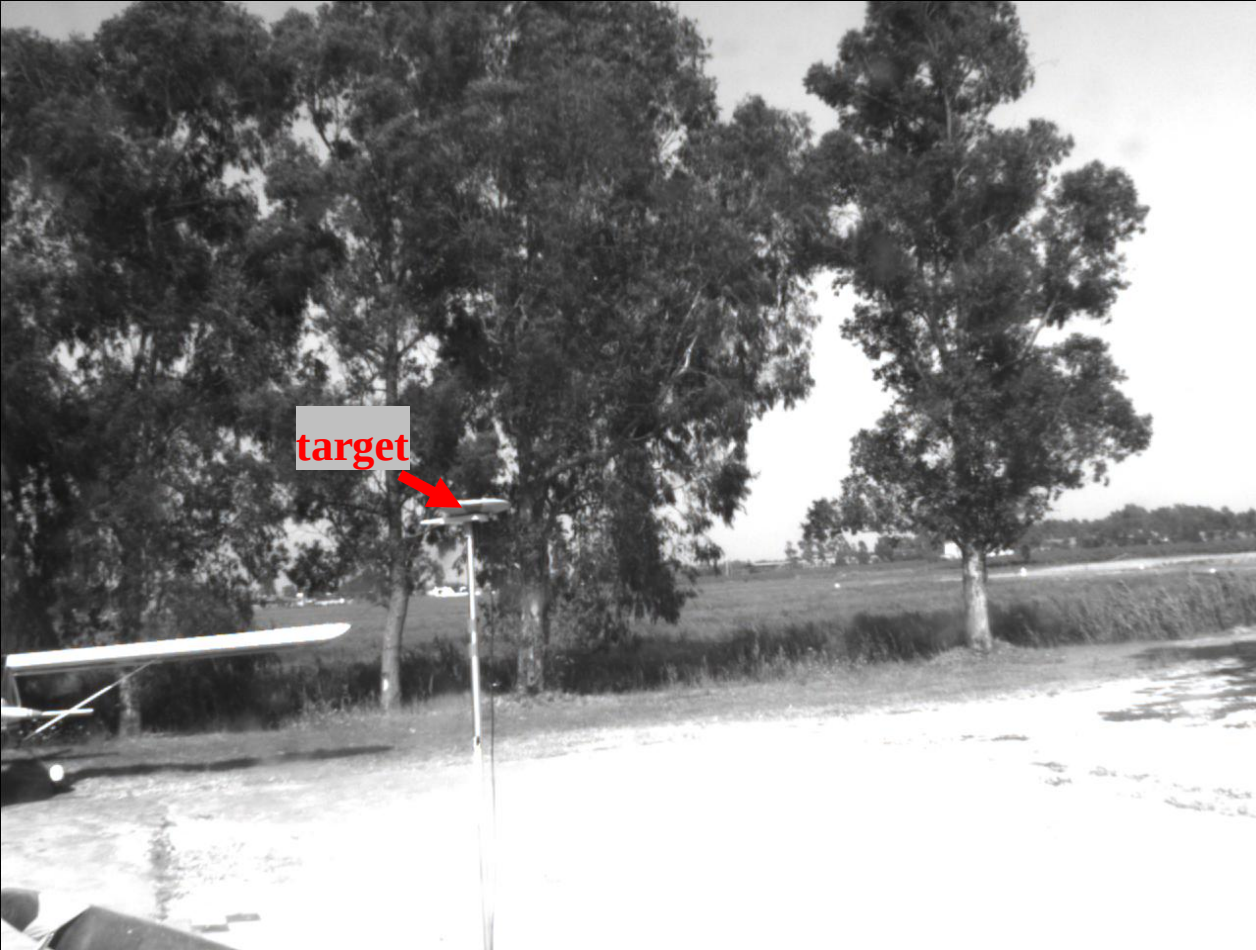
Example of panchromatic image taken during the alignment session.

**Figure 6.**
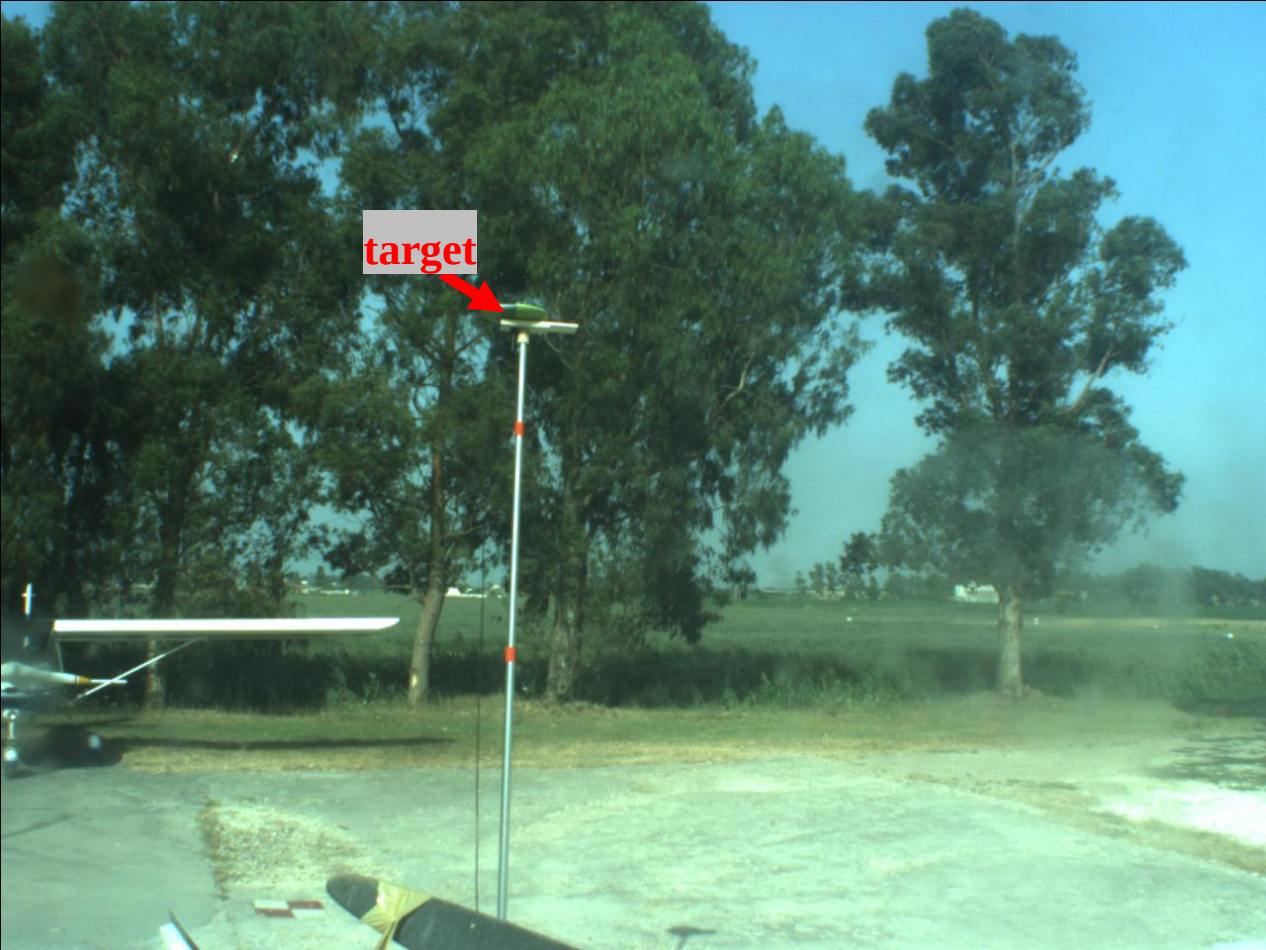
Example of color image taken during alignment session.

**Figure 7.**
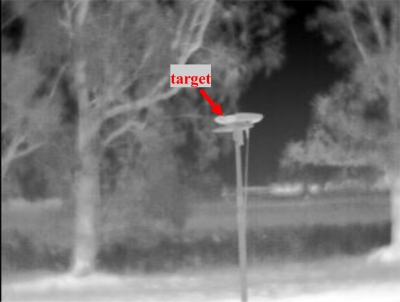
Example of thermal infrared image taken during alignment session.

Figures 5, 6, and 7 show some of the target images taken during the alignment tests by the panchromatic, the color and the IR cameras. It can be seen that target contrast with respect to the background allows for precise target pixel extraction in all the images.

**Figure 8.**
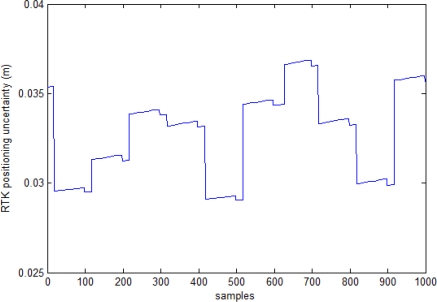
Auto-estimated uncertainty of target position estimation in a typical case.

The RTK technique allows one to estimate the level of relative positioning uncertainty during acquisitions [17]. A typical trend of this uncertainty is reported in Figure 8. The diagram refers to a certain target position and is comprised of 1,000 position samples. In the mean, the estimated uncertainty is on the order of about 3.5 cm which correspond to an angle of 0.5° at a distance of 4 m. Variation in attitude angles during acquisitions was on the order of 0.01° for roll and pitch, while the heading angle showed a more noisy trend with typical variations on the order of 0.1°. Output of alignment calculations is reported in Table 3.

**Figure 9.**
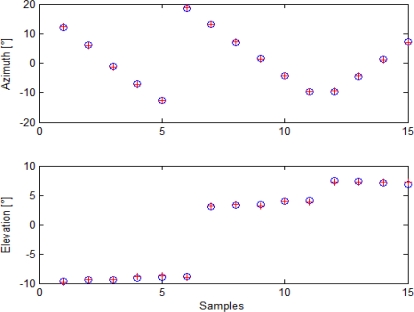
Target angular positions as extracted from images (blue circles) and from GPS/AHRS measurements and computed rotation matrix (red crosses) for the panchromatic camera.

**Figure 10.**
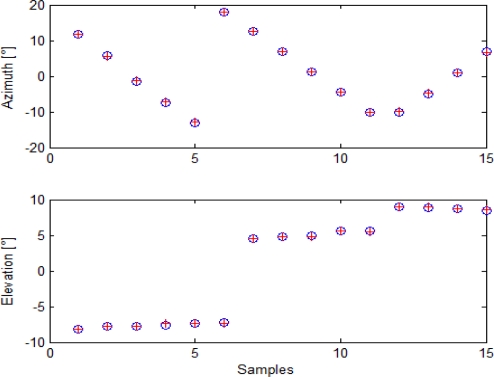
Target angular positions as extracted from images (blue circles) and from GPS/AHRS measurements and computed rotation matrix (red crosses) for the color camera.

**Figure 11.**
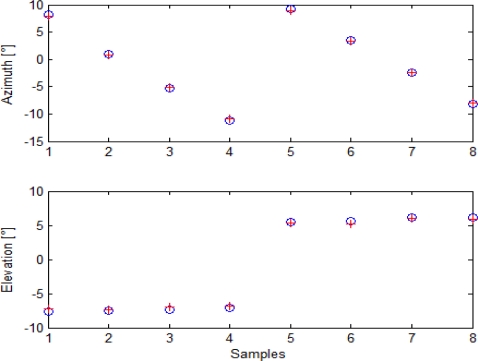
Target angular positions as extracted from images (blue circles) and from GPS/AHRS measurements and computed rotation matrix (red crosses) for the infrared camera.

Given the Euler angle estimates for the cameras, it is possible to analyze boresighting errors in order to evaluate uncertainty in pointing estimation. First of all, it is possible to compare target angular positions as reconstructed from GPS/AHRS measurements and Euler angles with the ones extracted by analysis in cameras’ images. This is done in Figure 9 for the panchromatic camera, in Figure 10 for the color camera, and in Figure 11 for the infrared camera.

As it can be seen, alignment calculation was based on 15 points for the visible cameras and on eight points for the infrared camera, due to its narrower field of view. Indeed, more target points were acquired during this alignment session, but post processing analysis of attitude data revealed that in some cases attitude estimation was affected by a higher noise, so those acquisitions were discarded to prevent from loss of precision. These diagrams offer a first proof of accuracy in cameras attitude estimation.

**Figure 12.**
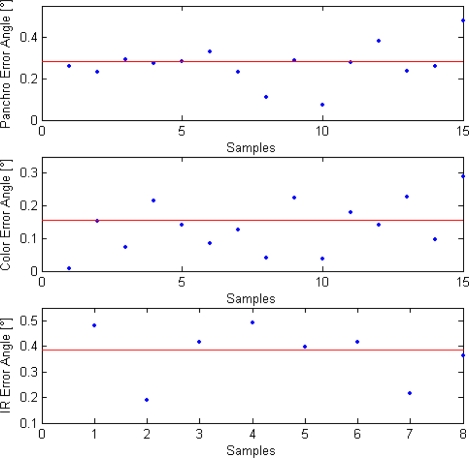
Angular errors for the different cameras. The red line represents RMS value.

Further insight into boresighting uncertainty is provided by Figure 12, which reports the angle between the target unit vector as measured from images and the same vector as generated from GPS/AHRS and estimated attitude. The rms value of this angle over all the samples can be taken as a measurement of alignment uncertainty. It can be seen that the best results are obtained for the color camera, with an rms error of about 0.16°. Same analysis for the panchromatic camera reveals an rms error of about 0.28°. The difference between these two errors can be explained by the better GPS accuracy that was estimated during color camera position acquisition. Finally, the rms error for the infrared camera is slightly worse (0.37°) which has a statistical origin since it is due to the availability of less measurements because of the narrower FOV. These results are consistent with the target positioning uncertainties shown before, the number of independent acquisitions, and the estimated uncertainties in camera position and aircraft attitude.

**Figure 13.**
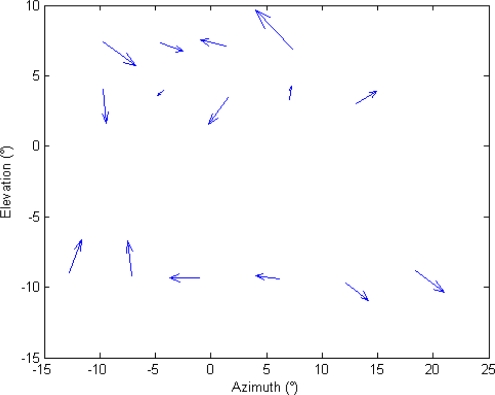
Vector representation of (magnified) azimuth and elevation residuals.

Finally, the random nature of these errors can be shown by means of a vectorial representation as in Figure 13. In this diagram, the differences between azimuth and elevation measured in the images and estimated *a posteriori* are plotted as the two components of a vector (after 10 times amplification). It can be seen that no systematic error pattern can be identified in the diagram.

## 5. Conclusions

This paper has focused on an accurate procedure for boresighting electro-optical sensors onboard aircraft and, in particular, Unmanned Aerial Vehicles. Relevant assumptions, mathematical methods and practical implementation issues were discussed. The presented procedure was applied to determine the alignment of EO sensors with respect to aircraft body-fixed axes for collision avoidance experiments carried out on the flying laboratory of the Italian Aerospace Research Center. The procedure is based on correlation of coordinates of targets identified in the EO images and of their positions and azimuth and elevation angles with respect to the aircraft measured by using GPS RTK and strapdown inertial systems. First experimental results showed that in the considered configuration the accuracy of the method is of the order of 0.1°, which is acceptable for the anti-collision application. Error analysis revealed that key factors for improving alignment accuracy are the stability of attitude and position estimates by AHRS and RTK differential GPS.

Noise in attitude estimation in static conditions is basically connected to the quality (hence cost) of the inertial navigation unit on board. Thus, in practical cases this parameter is dependent on the class of UAV or manned aircraft under consideration. It is also important to note that in general the absolute uncertainty in AHRS measurements is included in boresighting uncertainty. If alignment has to be realized with respect to the AHRS-defined reference frame, in a general case this uncertainty is unavoidable since the orientation of the AHRS-defined reference frame is by itself uncertain. However, it can be reduced by AHRS calibration. As for target positioning accuracy, impact of RTK accuracy on alignment quality can be improved by locating the target necessary for the procedure at a longer distance from the aircraft, which has to be traded off against simplicity of implementation.

In any case, it is worth noting that the developed technique allows for order of 0.1° alignment of EO sensors with respect to the navigation unit even in the case of low cost sensors and small platforms. This enforces the interest for EO cameras as obstacle detection sensors capable of providing very accurate obstacle angular information with respect to the aircraft, necessary to the collision avoidance decision-making logic, both for small and for large UAVs.

## Figures and Tables

**Table 1.** Camera accuracies and fields of view.

Measurement Precision of GPS TOPCON Legacy-H in carrier-phase mode	3 mm + 1 ppm
Visibile cameras IFOV	0.041°
IR cameras IFOV	0.075°
Visible cameras FOV (at res. 1280 X 960)	48.6° (H) × 37.6° (V)
IR cameras FOV (for each camera)	24° (H) × 18° (V)

**Table 2.** Linear dimensions corresponding to FOV and IFOV at different distances.

Distance [m]	2	4	5	10	20
Width FOV VIS [m]	1.8	3.6	4.5	9.0	18.0
Height FOV VIS [m]	1.4	2.7	3.4	6.8	13.6
Width FOV IR [m]	8.5 × 10^−1^	1.7	2.1	4.2	8.5
Height FOV IR [m]	6.3 × 10^−1^	1.3	1.6	3.2	6.3
Length IFOV VIS [m]	1.4 × 10^−3^	2.9 × 10^−3^	3.6 × 10^−3^	7.2 × 10^−3^	1.4 × 10^−2^
Length IFOV IR [m]	2.6 × 10^−3^	5.2 × 10^−3^	6.5 × 10^−3^	1.3 × 10^−2^	2.6 × 10^−2^

**Table 3.** Estimated cameras attitude angles.

**Estimated Angle (°)**	**Panchromatic**	**Color**	**Infrared**
Yaw	−3.94	3.89	0.85
Pitch	0.06	−1.48	0.29
Roll	−1.06	−1.48	−1.27
